# Transporter-Mediated Drug–Drug Interactions with Oral Antidiabetic Drugs

**DOI:** 10.3390/pharmaceutics3040680

**Published:** 2011-10-12

**Authors:** Sabine Klatt, Martin F. Fromm, Jörg König

**Affiliations:** Institute of Experimental and Clinical Pharmacology and Toxicology, Clinical Pharmacology and Clinical Toxicology, Friedrich-Alexander-Universität Erlangen-Nürnberg, 91054 Erlangen, Germany

**Keywords:** oral antidiabetic drug, organic cation transporter (OCT), organic anion transporting polypeptide (OATP), multidrug and toxin extrusion protein (MATE), ABC (ATP-binding cassette) transporter, P-glycoprotein, drug-drug interaction

## Abstract

Uptake transporters (e.g., members of the SLC superfamily of solute carriers) and export proteins (e.g., members of the ABC transporter superfamily) are important determinants for the pharmacokinetics of drugs. Alterations of drug transport due to concomitantly administered drugs that interfere with drug transport may alter the kinetics of drug substrates. *In vitro* and *in vivo* studies indicate that many drugs used for the treatment of metabolic disorders and cardiovascular diseases (e.g., oral antidiabetic drugs, statins) are substrates for uptake transporters and export proteins expressed in the intestine, the liver and the kidney. Since most patients with type 2 diabetes receive more than one drug, transporter-mediated drug-drug interactions are important molecular mechanisms leading to alterations in oral antidiabetic drug pharmacokinetics with the risk of adverse drug reactions. This review focuses on uptake transporters of the SLCO/SLC21 (OATP) and SLC22 (OCT/OAT) family of solute carriers and export pumps of the ABC (ATP-binding cassette) transporter superfamily (especially P-glycoprotein) as well as the export proteins of the SLC47 (MATE) family and their role for transporter-mediated drug-drug interactions with oral antidiabetic drugs.

## 1. Introduction

Drug effects result from the interplay of multiple processes that influence drug absorption, metabolism and excretion as well as drug response. Although, in past decades, most studies have focused on the importance of drug metabolizing enzymes (e.g., cytochrome P450 monooxygenases), in recent years it became evident that, in addition, transport proteins located in distinct membrane domains are important for drug effects. Furthermore, since all metabolizing enzymes are located intracellularly, the uptake of drugs from the extracellular space across the plasma membrane into the cell is a prerequisite for subsequent metabolism. Generally, transport proteins can be subdivided into two major groups: uptake transporters, mediating the transport of substances and drugs from the outside into cells and export proteins responsible for the translocation of substances or drugs and drug metabolites out of cells.

**Figure 1.**
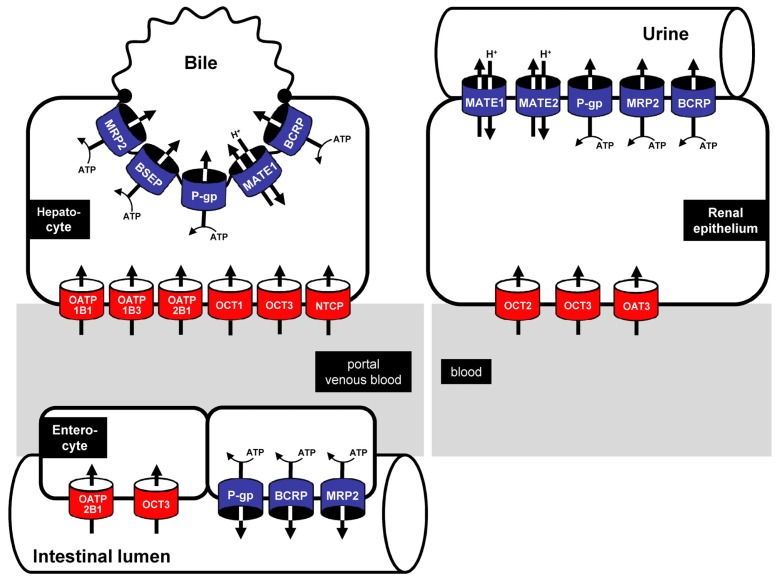
Uptake and export transporters involved in the intestinal absorption (enterocyte) and the hepatic (hepatocyte) and renal (renal epithelium) excretion of oral antidiabetic drugs. Uptake transporters (red): OATP = members of the organic anion transporting polypeptide family; OCT = members of the organic cation transporters; export transporters (blue): MRP2 = multidrug resistance protein 2; BSEP = bile salt export pump; BCRP = breast cancer resistance protein; P-gp = P-glycoprotein; MATE = members of the multidrug and toxin extrusion protein family.

Uptake transporters mostly belong to the superfamily of solute carriers (SLCs [1]). Today, the SLC superfamily is comprised of 378 different transport proteins grouped into 51 different transporter families. For detailed information on the SLC superfamily see [2]. Important transporter families within the SLC transporter superfamily are the SLC21/SLCO family, the SLC22 family and the SLC10 family. Organic anion transporting polypeptides (OATPs) are members of the SLC21/SLCO family [3–7] whereas organic cation transporters (OCTs) and organic anion transporters (OATs) are members of the SLC22 family [8]. This review describes oral antidiabetic drugs and transporter-mediated drug-drug interactions and we especially focus on the uptake transporters OATP1B1 (gene symbol: *SLCO1B1*), OATP1B3 (*SLCO1B3*) and OATP2B1 (*SLCO2B1*) of the SLC21/SLCO family, the SLC22 family members OCT1 (*SLC22A1*), OCT2 (*SLC22A2*) and OCT3 (*SLC22A3*) and the sodium-dependent bile salt transporter NTCP belonging to the SLC10 family. In human hepatocytes (Figure 1), the OATP family members OATP1B1 and OATP1B3 are predominantly, if not exclusively, expressed [9–11]. OATP2B1, the third highly expressed hepatic OATP, is also expressed in enterocytes (Figure 1) of the intestinum [12] and in several other tissues such as the heart [13]. The SLC22 family members OCT1 and OCT3 [14–16] as well as the SLC10 family member NTCP are also expressed in hepatocytes [17]. In addition, the presence of OCT2 and OCT3 in renal epithelial cells has been demonstrated (Figure 1; [18]).

Substrates for OATP1B1, OATP1B3 and OATP2B1 include several endogenously synthesized organic anions such as bile salts and hormone metabolites as well as widely prescribed drugs like HMG-CoA-reductase inhibitors (statins, e.g., atorvastatin [19–21]) and antibiotics (e.g., benzylpenicillin [22]). Physiological substrates of OCTs include hormones (e.g., corticosterone [23]) and neurotransmitters (e.g., epinephrine [23]) whereas OAT3 transports cAMP [24] and cGMP [25]. Drugs transported by OCTs include antineoplastic agents (cisplatin [26,27]) as well as the antidiabetic drug metformin [28]. Interestingly, the sodium-dependent bile salt transporter NTCP also transports drugs as it has been demonstrated for the HMG-CoA-reductase inhibitor rosuvastatin [29].

Efflux transporters mainly belong to the ATP-binding cassette (ABC) transporter family [30–33]. These export pumps use energy derived from ATP hydrolysis to mediate substrate transport, often against a concentration gradient. Today, the human ABC superfamily consists of 49 different ABC transporters grouped into 6 different ABC families [ABCA–ABCG (for detailed information see [34]). In the focus of the review are the ABCB family member P-glycoprotein (P-gp, *ABCB1*, [35]), the bile salt export pump BSEP (*ABCB11*) and the ABCG family member BCRP (*ABCG2*). P-gp and BCRP are expressed in enterocytes, hepatocytes and renal epithelial cells (Figure 1; [36–38]), whereas the expression of BSEP is restricted to hepatocytes (Figure 1; [39]).

P-gp is the best characterized human drug transporter with a very broad substrate spectrum transporting a variety of different drugs [40–42]. The ABCG family member BCRP, transports several endogenous compounds and is further capable of transporting a variety of different drugs including statins, calcium channel blockers and antivirals [43].

The MATE transporter family (multidrug and toxin extrusion; gene symbol *SLC47*) consists of two members, MATE1 (*SLC47A1*) and MATE2 [MATE2-K (*SLC47A2*)], which are localized to apical membrane domains. Whereas human MATE1 is strongly expressed in liver and kidney (Figure 1) and to a lesser extent in several other tissues including skeletal muscle and testis [44,45], MATE2 is almost exclusively expressed in the kidney and localized in the luminal membrane of proximal tubular epithelial cells. In contrast to the ATP-driven ABC transporters, MATE proteins are electroneutral transporters using an oppositely directed proton gradient as driving force (Figure 1). MATE1 and MATE2 have similar substrate and inhibitor specificities which overlap with those of OCTs. Endogenous substrates include organic cations (e.g., creatinine, guanidine) as well as clinically used drugs such as the antimalarial drug quinine [45,46], the antineoplastic agent cisplatin [27] and the antidiabetic drug metformin [46]. Detailed information on tissue distribution and substrate spectrum of the respective uptake and export transporters are summarized in the mentioned reviews [8,14,33,47].

After administration and passage through the intestine, oral antidiabetic drugs have to be taken up from the portal venous blood via the basolateral membrane into hepatocytes before they are metabolized, cause drug effects via intrahepatic mechanisms or are transported back into the systemic circulation for extrahepatic effects. So far, little is known on the involvement of oral antidiabetic drugs into intestinal transporter-mediated drug-drug interactions [48]. The characteristics of some widely used oral antidiabetic drugs together with their mode of action, metabolizing enzymes and transport proteins relevant for their membrane translocation or drug-drug interactions are summarized in Table 1. Since patients with type 2 diabetes are commonly treated with more than one drug (in most cases one or more oral antidiabetic drug and additionally a statin and an antihypertensive drug), it is essential to understand the molecular mechanisms underlying drug-drug interactions, which might cause changes in the effect or the pharmacokinetics of these drugs. Aside from metabolizing enzymes [49–52] it is now well established that also modification of transport function is involved in these drug-drug interactions. Therefore, the role of transport proteins for drug-drug interactions with frequently used oral antidiabetic drugs presented in table 1 are in the focus of this review.

## 2. Oral Antidiabetic Drugs and OATPs

**Figure 2.**
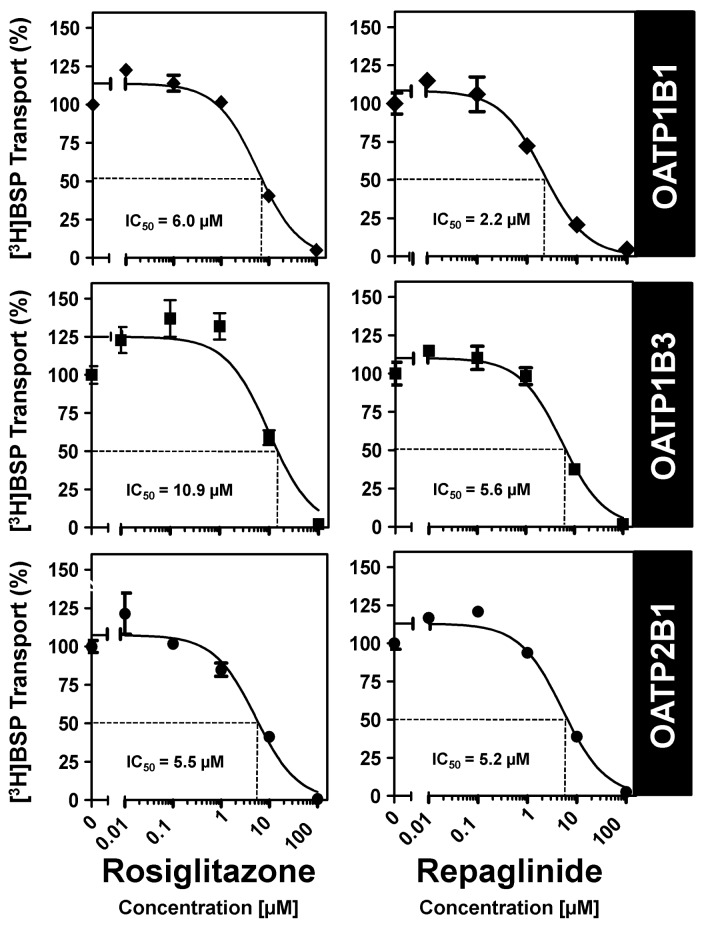
Inhibition of OATP1B1- (top), OATP1B3- (middle) and OATP2B1-mediated (bottom) BSP uptake by the oral antidiabetic drugs rosiglitazone (left) and repaglinide (right). Data are shown as the percentage of BSP uptake (0.05 μM BSP for OATP1B1- and 1 μM BSP for OATP1B3- and OATP2B1-mediated transport; modified from [64]) in the absence of the respective drug.

**Figure 3.**
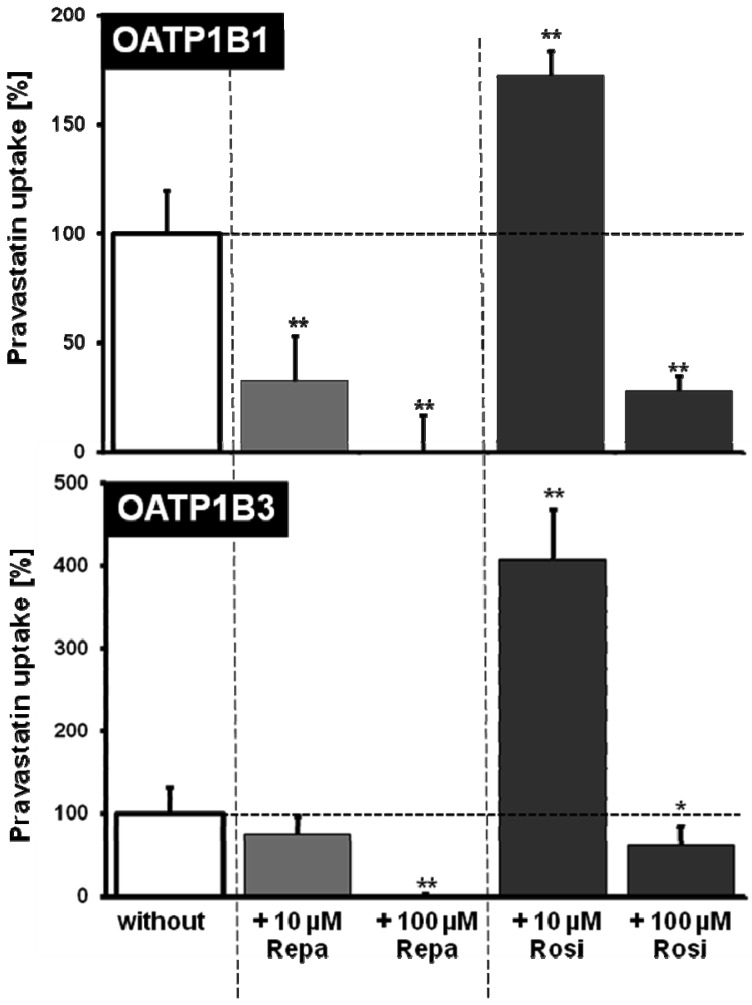
Inhibition of OATP1B1- (top) and OATP1B3-mediated (bottom) pravastatin uptake (50 μM) by the oral antidiabetic drugs repaglinide (Repa) and rosiglitazone (Rosi). Data are shown as the percentage of OATP1B1- or OATP1B3-mediated pravastatin uptake in the absence of the respective drug (without). *P < 0.05; **P < 0.01 *vs.* control. (modified from [64]).

OATP1B1, OATP1B3 and OATP2B1 are localized in the basolateral membrane transporting substances and drugs from the portal venous blood into hepatocytes [4,47]. OATP2B1 is further expressed in enterocytes and localized there to the luminal membrane mediating the uptake of substances and drugs from the intestinal lumen into the body [12]. Several drugs have been identified as substrates for these OATPs including antibiotics and HMG-CoA-reductase inhibitors (statins) [5,10]. Repaglinide was one of the first oral antidiabetic drugs shown to interact with hepatic OATPs. More indirect evidence for the involvement of OATP1B1 in repaglinide pharmacokinetics has been published by Niemi and colleagues [52]. In this study they investigated the *in vivo* effect of cyclosporine, a known inhibitor of CYP3A4 and OATP1B1 on the pharmacokinetics and pharmacodynamics of repaglinide. They found that cyclosporine raised the plasma concentrations of concomitantly administered repaglinide probably by inhibiting its OATP1B1-mediated hepatic uptake and the subsequent metabolism by CYP3A4. In the same year the authors published a second *in vivo* study [63] demonstrating that a polymorphic variant of the OATP1B1 protein is a major determinant of the interindividual variability in the pharmacokinetics of repaglinide. Interestingly, polymorphic variants of the OATP1B1 protein seem not to have a significant effect on nateglinide, the second frequently used meglitinide derivative [66] and did not influence the pharmacokinetics of rosiglitazone or pioglitazone [90]. Based on these observations, Bachmakov *et al.* investigated the effect of repaglinide and rosiglitazone on OATP1B1-, OATP1B3- and OATP2B1-mediated transport *in vitro* [64]. Using stably transfected HEK cells recombinantly overexpressing these OATP family members and BSP as prototypic substrate, they found that both rosiglitazone and repaglinide inhibited the uptake mediated by these three transporters (Figure 2). Both oral antidiabetic drugs showed a potent uptake inhibition with IC_50_ values around 10 μM. In the same study they also used pravastatin as substrate for OATP1B1 and OATP1B3 and they demonstrated that repaglinide at a concentration of 10 μM significantly inhibited OATP1B1-mediated pravastatin uptake, whereas the pravastatin uptake by OATP1B3 was only slightly reduced and significantly inhibited only at a repaglinide concentration of 100 μM (Figure 3). An interesting observation was made analyzing the effect of rosiglitazone on OATP1B1- and OATP1B3-mediated pravastatin uptake. Whereas repaglinide at a concentration of 10 μM inhibited the uptake, rosiglitazone at the same concentration stimulated pravastatin uptake by OATP1B1 and OATP1B3 and at the same time shows inhibition only at the highest tested concentration of 100 μM (Figure 3). This demonstrated that not only uptake inhibition but also stimulation of drug uptake could be a result of drug-drug interactions. Recently, this stimulatory effect has been analyzed in detail using pravastatin as substrate and non-steroidal anti-inflammatory drugs as interacting substances [91]. In this study the authors showed that ibuprofen stimulated OATP1B1- and OATP1B3-mediated pravastatin uptake likely by an allosteric mechanism without being transported. In the case of rosiglitazone one can assume that at low concentrations the uptake of pravastatin can be stimulated by acting as allosteric modulator and that only at high substrate concentrations a competitive inhibition can be detected. However, the clinical relevance of these findings remains to be clarified.

Glibenclamide is a frequently prescribed insulin secretagogue stimulating the insulin secretion from pancreatic β-cells. It is extensively metabolized by CYP2C9, CYP2C19 and CYP3A4 [53,55] and seems to be a substrate for OATP2B1 [54]. Using HEK cells overexpressing OATP2B1 Satoh *et al.* have shown that grapefruit and citrus juice inhibited OATP2B1-mediated glibenclamide uptake [54]. Since OATP2B1 is also localized in the apical membrane of enterocytes this may result in a reduced intestinal absorption of orally administered glibenclamide and of other drugs that are substrates of OATP2B1. Interestingly, this could not be confirmed in an *in vivo* study analyzing the effect of clarithromycin and grapefruit juice on the pharmacokinetics of glibenclamide [92]. In this study 12 subjects ingested 250 mg clarithromycin or placebo with 200 mL grapefruit juice three times daily. On day three, they ingested in addition 0.875 mg glibenclamide with sugar water or grapefruit juice and the concentrations of glibenclamide and clarithromycin in plasma, glucose in blood and excretion of the metabolite hydroxyglibenclamide in urine were measured. These analyses demonstrated that clarithromycin increased plasma concentrations of glibenclamide, maybe by inhibiting OATP2B1-mediated uptake and CYP3A4 metabolism in the intestine, but no effect of grapefruit juice on glibenclamide pharmacokinetics could be detected. The results of this study were confirmed in a study by Niemi *et al.* demonstrating that clarithromycin also increases the plasma concentrations and effects of simultaneously administered repaglinide [49].

An interaction of atorvastatin with glibenclamide has been demonstrated using MDCK cells stably expressing OATP2B1 [13]. In this study the authors investigated the expression of OATP2B1 in human heart samples and analyzed OATP2B1-mediated atorvastatin and glibenclamide transport and transport inhibition by simultaneously administered drugs. They demonstrated that OATP2B1-mediated glibenclamide transport was inhibited not only by atorvastatin but also by simvastatin, cerivastatin and estrone-3-sulfate (E3S), whereas OATP2B1-mediated E3S uptake was inhibited by gemfibrozil. A potential hazardous interaction between gemfibrozil and repaglinide has been described in 2003 by Niemi and coworkers [50]. It was shown that this interaction persists for at least 12 h after administration of gemfibrozil [93] suggesting that not only the inhibition of uptake transporters but also the inhibition of the drug metabolizing enzyme CYP2C8 might by important for this drug-drug interaction. Another study investigated the effects of atorvastatin on repaglinide pharmacokinetics in relation to *SLCO1B1* polymorphism and it could be demonstrated that atorvastatin raises repaglinide plasma concentration, probably by inhibiting OATP1B1 [94]. Other OATP-related drug-drug interactions with oral antidiabetic drugs are summarized in Table 2. Taken together, these analyses revealed that OATPs are important molecular targets of transporter-mediated drug-drug interactions since their substrate spectrum includes a variety of frequently prescribed drugs often administered concomitantly with oral antidiabetic drugs.

## 3. Oral Antidiabetic Drugs and OCTs

OCTs are members of the SLC22 family expressed in several tissues including intestine, liver and kidney (Figure 1; [8]). Since a large number of clinically used drugs are administered orally, of which approximately 40% are cations or weak bases at physiological pH [119], transport proteins for cations are important determinants of drug pharmacokinetics. Several antidiabetic drugs have been identified as substrates or inhibitors of OCT proteins. These include metformin which is a substrate for OCT1 (*K*_m_ value = 1 470 μM [82]), OCT2 (*K*_m_ value = 990 μM [82]) and OCT3 (*K*_m_ value = 2 260 μM [15]), whereas phenformin [120], repaglinide [121] and rosiglitazone [64] all interact with OCT1. Furthermore, genetic variations in the *SLC22A1* gene encoding OCT1 were associated with altered pharmacokinetics and pharmacodynamics of metformin [122,123], which might pose a higher risk for side effects of metformin, especially lactic acidosis ([86], for review see [124]). Several OCT-mediated drug-drug interactions have been described using either metformin as a substrate for OCTs or other oral antidiabetic drugs inhibiting OCT-mediated transport. *In vivo* it has been shown that genetic variations in the *SLC22A2* gene encoding human OCT2 are associated with alterations in metformin pharmacokinetics and that in the presence of simultaneously administered cimetidine the renal clearance of metformin was decreased [108,109]. This uptake inhibition could be confirmed *in vitro* using HEK and MDCK cells overexpressing OCT1 [28] and OCT2 [110,111]. For OCT1, the IC_50_ value for cimetidine-induced inhibition of metformin uptake was 150 μM [28], for OCT2 a *K*_i_ value of 147 μM could be detected for the same drug combination [111]. Other drugs inhibiting OCT-mediated metformin uptake are the BCR-ABL inhibitors imatinib and nilotinib [112]. Imatinib inhibited the uptake with IC_50_ values of 1.5 μM, 5.8 μM and 4.4 μM for OCT1, OCT2 and OCT3, respectively whereas nilotinib inhibited the same transporters with IC_50_ values of 2.9 μM (OCT1), >30 μM (OCT2) and 0.3 μM (OCT3). Furthermore, in the same study the inhibition of OCT-mediated metformin uptake by the EGFR (epidermal growth factor receptor) inhibitor gefitinib was studied demonstrating an inhibition of OCT1, OCT2 and OCT3 with IC_50_ values of 1.1 μM, 24.4 μM and 5.5 μM, respectively.

Since the uptake of drugs from blood into the renal tubular cells is a key determinant for renal secretion, inhibition of OCT2-mediated drug transport may influence systemic plasma concentration. For beta-blockers this uptake inhibition has been demonstrated *in vitro* using MDCK cells recombinantly overexpressing OCT2. In this study, Bachmakov and coworkers showed that OCT2-mediated metformin uptake was significantly inhibited by the beta-blockers bisoprolol, carvedilol, metoprolol and propranolol with IC_50_ values of 2.4 μM, 2.3 μM, 50.2 μM and 8.3 μM, respectively [106].

Interestingly, the uptake and the effect of one oral antidiabetic drug can also be inhibited by a second antidiabetic drug. This has been demonstrated *in vitro* analyzing OCT1- and OCT2-mediated metformin uptake and uptake inhibition by the DPP-4 inhibitor sitagliptin [87]. In this study the authors showed that sitagliptin inhibited OCT1- and OCT2-mediated metformin uptake with IC_50_ values of 34.9 μM and 40.8 μM, respectively. Furthermore, the inhibition of metformin-induced activation of AMPK (5′ adenosine monophosphate-activated protein kinase) signaling was investigated demonstrating that treatment with sitagliptin in MDCK-OCT1 and HepG2 cells resulted in a reduced level of phosphorylated AMPK with K_i_ values of 38.8 μM and 43.4 μM, respectively.

Summarizing these data (in combination with data presented in Table 2) one can assume that, in addition to OATP-mediated interactions, the inhibition of OCT proteins may be an important determinant of oral antidiabetic drug pharmacokinetics.

## 4. Oral Antidiabetic Drugs and Export Proteins

Most export proteins that mediate the export of drugs and drug metabolites out of cells belong to the superfamily of ABC transporters. P-glycoprotein (P-gp) is the best characterized efflux transporter localized in the apical membrane of enterocytes, the bile canalicular membrane and proximal tubule cells of the renal epithelium (Figure 1) mediating the export of a variety of different drugs belonging to various drug classes [35]. In diabetes therapy, several *in vivo* and *in vitro* studies have shown that P-gp is also involved in drug-drug interactions. It has been demonstrated *in vivo* that the macrolide clarithromycin increased the C_max_ and the AUC (area under the plasma concentration time curve) of coadministered glibenclamide [92] and thus could lead to hypoglycaemia due to elevated plasma concentrations of glibenclamide [60]. Further P-gp-mediated drug-drug interactions could be observed in several *in vitro* studies. Using P-gp overexpressing cells it could be demonstrated that glibenclamide inhibited P-gp-mediated efflux of the model substrate rhodamine 123 [57]. Inhibition of glibenclamide export resulting in increased intracellular accumulation was also detected in the presence of colchicine [56]. Another study conducted by Hemauer and coworkers showed that rosiglitazone and metformin were transported by P-gp using placental inside-out oriented brush border membrane vesicles. Additionally, it was reported that inhibition of P-gp by verapamil markedly decreased the transport of rosiglitazone and metformin [72].

A second well-studied export pump involved in the transport of oral antidiabetic drugs is the breast cancer resistance protein BCRP (gene symbol *ABCG2*). This transporter is localized in the apical membrane of enterocytes and renal epithelial cells as well as in the canalicular membrane of hepatocytes (Figure 1). Several *in vitro* studies have indicated that oral antidiabetic drugs are substrates for BCRP and that coadministration of other drugs may inhibit this BCRP-mediated transport. Like for P-gp, Hemauer *et al.* provided evidence that BCRP participates in the transport of metformin. They could show that the BCRP inhibitor KO143 lead to a decreased metformin transport [72]. In addition, several studies using cells stably expressing BCRP revealed that glibenclamide export is inhibited by prototypic inhibitors, such as the antibiotic novobiocin or fumitremorgin C [57,97]. Pollex *et al.* further investigated the functional consequences of a frequent genetic variation in the *ABCG2* gene encoding human BCRP. They found that the variation BCRPp.Q141K (ABCG2c.412C>A) showed a higher Michaelis Menten constant (*K*_m_ value) and a higher maximal transport rate (*V*_max_ value) for BCRP-mediated glibenclamide transport as compared to the transport mediated by the wild-type BCRP protein [97].

Interesting and recently characterized transporters responsible for the export of substances and drugs out of cells are members of the MATE family (multidrug and toxin extrusion protein; gene symbol *SLC47*). As known so far, this family consists of the two members MATE1 [125] and MATE2 (also designated as MATE2-K [45]). MATE1 is expressed in several tissues including liver and kidney, whereas expression of MATE2 seems to be restricted to renal epithelial cells (Figure 1; [44]). Unlike ABC transporters, these export proteins use an inward-directed proton gradient for exporting substrates out of cells. Several endogenous substances and drugs have been identified as substrates of these transport proteins [126] with metformin as the best studied oral antidiabetic drug transported by MATE1 and MATE2 [46]. The importance of MATE1 for the pharmacokinetics of metformin has been investigated in Mate1 (−/−) knockout mice [127]. After a single intravenous administration of metformin (5 mg/kg), a two-fold increase in the AUC of metformin in Mate1 (−/−) mice as compared to wild-type mice was detected. Furthermore, the renal clearance of metformin was only 18% of that measured in Mate1 (+/+) mice clearly demonstrating the essential role of MATE1 in the systemic clearance of metformin.

Inhibition studies using single-transfected HEK cells recombinantly expressing MATE1 or MATE2 showed that some tyrosine kinase inhibitors (TKIs) are potent inhibitors of MATE-mediated transport [112]. In detail, imatinib, nilotinib, gefitinib and erlotinib inhibited MATE1- and MATE2-mediated metformin transport with unbound C_max_/IC_50_ values higher than 0.1, suggesting that this transporter-mediated drug-drug interaction with TKIs may also be relevant *in vivo* and therefore affect the disposition, efficacy and toxicity of metformin and possibly of other drugs that are substrates for these export proteins. A further analysis of drug interaction studies has been performed by zu Schwabedissen and coworkers [105]. They demonstrated a significant inhibition of MATE1-mediated metformin transport by using single-transfected HeLa cells expressing human MATE1 and a panel of 24 different drugs. Most potent inhibitors (all tested at a concentration of 25 μM) were cimetidine with 21.08 ± 0.81%, trimethoprim with 25.35 ± 0.49% and ritonavir with 25.75 ± 9.91% residual metformin transport. Furthermore, the IC_50_ values of MATE1-mediated metformin transport were determined for ritonavir (15.4 ± 2.5 μM), ranitidine (18.9 ± 7.3 μM), rapamycin (3.27 ± 0.46 μM) and mitoxantrone (4.4 ± 1.3 μM). All of these IC_50_ values are below the reported peak plasma concentrations, suggesting that these interactions may also play a role *in vivo* [105]. Another interaction of MATE1-mediated metformin uptake has been analyzed in HEK cells stably expressing MATE1 and the frequently prescribed antibiotic trimethoprim as well as the antimalarial drug chloroquine. The experiments revealed a strong inhibition for both substances with an IC_50_ value of 6.2 μM calculated for trimethoprim and a K_i_ value of 2.8 μM for chloroquine [107].

Some studies also investigated the effect of drugs on OCT- and MATE-mediated transport using double-transfected cells recombinantly expressing an uptake transporter of the OCT family together with MATE1 or MATE2 [128,129]. In order to examine the effect of cimetidine on transcellular metformin transport, an OCT2-MATE1 double-transfected MDCK cell line was used [111]. These experiments demonstrated that the transcellular metformin transport was moderately inhibited by 1 μM cimetidine and almost completely inhibited by 1 mM cimetidine. Interestingly, the intracellular accumulation of metformin was inhibited only by 1 mM cimetidine but was increased by the low concentration of 1 μM cimetidine under the same experimental conditions. These results suggest that low cimetidine concentrations had no inhibitory effect on OCT2-mediated uptake whereas MATE1 was inhibited by intracellular cimetidine resulting in an increased intracellular metformin concentration. Taken together, these studies demonstrated that beside the inhibition of uptake transporters also the inhibition of export proteins may be important and clinically relevant for drug-drug interactions with oral antidiabetic drugs. Furthermore, experimental approaches using double-transfected cell lines simultaneously expressing an uptake transporter and an export protein indicated a crucial interaction of uptake transporters and export pumps.

## Figures and Tables

**Table 1.** Characteristics of selected oral antidiabetic drugs.

**Oral antidiabetic drugs**	**Characteristics**	**Action**	**Elimination#**	**Metabolism**	**Transporters+**	**References**

Glibenclamide	Sulfonylurea derivatives, insulin secretagogues	Stimulation of insulin secretion from pancreatic β-cells by binding to ATP-dependent potassium channels Side effects: weight gain, hypoglycaemia	Renal (50%)^a^ Biliary (50%)^a^	CYP2C9 CYP2C19 CYP3A4	OATP2B1 P-gp BCRP	[53,54] [55,56] [55,57]
Glimepiride			Renal (60%)^b^ Biliary (40%)^b^	CYP2C9	-	[58,59] [60,61]*

Repaglinide	Meglitinide derivatives, insulin secretagogues	Stimulation of insulin secretion from pancreatic ß-cells by binding to ATP-dependent potassium channels Side effects: mild hypoglycaemia^c^, ^d^	Renal (<8%)^c^ Biliary (90%)^c^	CYP2C8 CYP3A4	OATP1B1 OATP1B3	[62,63] [62,64]
Nateglinide			Renal (83%)^d^ Biliary (10%)^d^	CYP2C9 CYP3A4	-	[65,66] [65] [67]*

Rosiglitazone	Tiazolidine-diones, insulin sensitizers	Peroxisome proliferator-activated receptor γ agonists (PPAR) Side effects: fluid retention, increased incidence of heart failure and fracture risk; hepatotoxicity (troglitazone)	Renal (65%) Biliary (30%) [68]	CYP2C8 CYP2C9	OATP1B1 OATP1B3 BCRP P-gp	[64,70] [58,64] [71] [72]
Pioglitazone			Renal (45%)^e^ Biliary (55%)^e^	CYP2C8 CYP3A4	OATP1B1 OATP1B3	[73,74] [58,74]
Troglitazone			Renal (3%) Biliary (85%) [69]	CYP3A4 CYP2C8	OATP1B1 OATP1B3 BCRP P-gp BSEP	[58,74] [58,75] [76] [76] [77] [78–81]*

Metformin	Biguanide	Activation of AMP-activated protein kinase (AMPK) and suppression of glucagon-stimulated glucose production, increase in glucose uptake in muscle and hepatic cells Side Effect: lactic acidosis	Biliary (100%)^f^	-	OCT1-3 MATE1-2	[15,82] [46,58] [83–86]*

Sitagliptin	Dipeptidyl peptidase-4 inhibitor (DDP-4)	Prolongation of Glucagon-like peptide 1 (GLP-1) action by inhibition of DPP-4 Side effects: nausea, hypoglycaemia^g^	Renal (87%)^g^ Biliary (13%)^g^	-	OCT1-2 OAT3 P-gp	[87] [88] [88] [89]*

* References refer to general characteristics of the respective oral antidiabetic drug(s);

+ Oral antidiabetic drug is substrate and/or inhibitor of the respective transporter;

# according to the Summary of Product Characteristics—SPC (Fachinformation) if not stated otherwise;

a Glibenclamide (Euglucon) product information, Sanofi-Aventis GmbH, Frankfurt a. M., Germany, 2008

b Glimepiride (Amaryl) product information, Sanofi-Aventis GmbH, Frankfurt a. M., Germany, 2006

c Repaglinide (NovoNorm)product information, Novo Nordisk Pharma AG, Küsnacht, Switzerland, 2010;

d Nateglinide (Starlix) product information, Novartis Pharma GmbH, Nürnberg, Germany, 2009;

e Pioglitazone (Actos) product information, Takeda Pharma, Aachen, Germany, 2010;

f Metformin (Glucophage) product information, Merck Pharma GmbH, Darmstadt, Germany, 2006;

g Sitagliptin (Januvia) product information, MSD Shark & Dohme GmbH, Haar, Germany, 2010.

**Table 2.** Selected human solute carriers (SLC) and ATP-binding cassette (ABC) transporters involved in interactions with oral antidiabetic drugs.

**Oral antidiabetic drug**	**Inhibitor/victim compound**	***In vivo/in vitro***	**Effect**	**CYP**	**Transporters**	**Reference(s)**
Glibenclamide	5-CFDA	*in vitro*	Inhibition of 5-CFDA efflux	-	MRP1-3	[57]
	8-FcA	*in vitro*	Inhibition of 8-FcA uptake (IC_50_ = 1.1 and 2.7 μM)	-	OATP1B1, OATP1B3	[95]
	Atorvastatin	*in vitro*	Inhibition of atorvastatin uptake	-	OATP2B1	[13]
	Atorvastatin	*in vitro*	Inhibition of glibenclamide uptake	-	OATP2B1	[13]
	Calcein	*in vitro*	Inhibition of calcein efflux	-	MRP1	[96]
	Cerivastatin	*in vitro*	Inhibition of glibenclamide uptake	-	OATP2B1	[13]
	Clarithromycin	*in vivo*	C_max_ and AUC of glibenclamide ↑	CYP3A4	P-gp	[92]
	Colchicine	*in vitro*	Increased intracellular accumulation of colchicine	-	P-gp	[56]
	Estrone-3-sulfate	*in vitro*	Inhibition of E3S uptake	-	OATP2B1	[13]
	Estrone-3-sulfate	*in vitro*	Inhibition of glibenclamide uptake	-	OATP2B1	[13]
	Fumitremorgin C	*in vitro*	Increased intracellular accumulation of glibenclamide	-	BCRP	[97]
	GCDC	*in vitro*	Inhibition of GCDC transport (IC_50_ = 7.6 μM)	-	BSEP	[77]
	Ginkgo leaf extract	*in vitro*	Inhibition of glibenclamide uptake (IC_50_ = 15.4 μg/mL)	-	OATP2B1	[98]
	Glycocholate	*in vitro*	Inhibition of glycocholate transport (IC_50_ = 18.8 μM)	-	BSEP	[77]
	Grapefruit juice	*in vitro*	Inhibition of glibenclamide uptake	-	OATP2B1	[54]
	Grapefruit juice	*in vivo*	No effect on glibenclamide pharmacokinetics	-	-	[92]
	Green tea extract	*in vitro*	Inhibition of glibenclamide uptake (IC_50_ = 24.6 μg/mL)	-	OATP2B1	[98]
	Indomethacin	*in vitro*	Decreased glibenclamide uptake in IOVs	-	MRP1	[72]
	Indomethacin	*in vitro*	Increased intracellular accumulation of glibenclamide	-	MRP3	[57]
	KO143	*in vitro*	Decreased glibenclamide uptake in IOVs	-	BCRP	[72]
	Nicardipine	*ex vivo* a	Increase in mean fetal-to-maternal concentration ratio	-	BCRP	[99]
	Novobiocin	*in vitro*	Increased intracellular accumulation of glibenclamide	-	BCRP	[57,100]
	Orange juice	*in vitro*	Inhibition of glibenclamide uptake	-	OATP2B1	[54]
	Pitavastatin	*in vitro*	Inhibition of pitavastatin uptake	-	OATP1B1	[101]
	Rhodamine 123	*in vitro*	Inhibition of rhodamine 123 efflux	-	P-gp	[57]
	Rifampin	*in vivo*	AUC of glibenclamide ↑ 2.3-fold	-	OATP1B1	[102]
	Simvastatin	*in vitro*	Inhibition of glibenclamide uptake	-	OATP2B1	[13]
	Taurocholate	*in vitro*	Inhibition of taurocholate uptake	-	NTCP	[103]
	Taurocholate	*in vitro*	Increased intracellular accumulation of taurocholate	-	BSEP	[103]
	Taurocholate	*in vitro*	Inhibition of taurocholate transport (*K*_i_ = 27.5 μM)	-	BSEP	[104]
	Taurocholate	*in vitro*	Inhibition of taurocholate transport (IC_50_ = 14.7 μM)	-	BSEP	[77]
	TCDC	*in vitro*	Inhibition of TCDC transport (IC_50_ = 15.8 μM)	-	BSEP	[77]
	Verapamil	*in vitro*	Decreased glibenclamide uptake in IOVs	-	P-gp	[72]
Glimepiride	Gemfibrozil	*in vivo*	AUC of Glimepiride ↑ 23%	CYP2C9	-	[59]
Metformin	Amprenavir	*in vitro*	Inhibition of metformin uptake	-	OCT2, MATE1	[105]
	Bisoprolol	*in vitro*	Inhibition of metformin uptake (IC_50_ = 2.4 μM)	-	OCT2	[106]
	Carvidolol	*in vitro*	Inhibition of metformin uptake (IC_50_ = 2.3 μM)	-	OCT2	[106]
	Chloroquine	*in vitro*	Inhibition of metformin uptake (*K*_i_ = 2.8 μM)	-	MATE1	[107]
	Cimetidine	*in vivo*	Reduction of renal clearance	-	OCT2	[108,109]
	Cimetidine	*in vitro*	Inhibition of metformin uptake (IC_50_ = 11 μM)	-	OCT2	[110]
	Cimetidine	*in vitro*	Inhibition of metformin uptake (*K*_i_ = 147 μM)	-	OCT2	[111]
	Cimetidine	*in vitro*	Inhibition of metformin uptake (IC_50_ = 158 μM)	-	OCT1	[28]
	Cimetidine	*in vitro*	Inhibition of metformin uptake	-	OCT2, MATE1	[105]
	Cimetidine	*in vitro*	Inhibition of metformin uptake (*K*_i_ = 1.1 μM)	-	MATE1	[111]
	Dipyridamole	*in vitro*	Inhibition of metformin uptake	-	OCT2, MATE1	[105]
	Erlotinib	*in vitro*	Inhibition of metformin uptake			
			IC_50_ (μM) = 0.4 (OCT1), 5.2 (OCT2), 4.2 (OCT3)	-	OCT1-3	
			IC_50_ (μM) = 7.9 (MATE1), 3.5 (MATE2)	-	MATE1-2	[112]
	Fenfluramine	*in vitro*	Inhibition of metformin uptake	-	OCT2	[113]
	Gefitinib	*in vitro*	Inhibition of metformin uptake			
			IC_50_ (μM) = 1.1 (OCT1), 24.4 (OCT2), 5.5 (OCT3)	-	OCT1-3	
			IC_50_ (μM) = 1.8 (MATE1), 0.2 (MATE2)	-	MATE1-2	[112]
	Imatinib	*in vitro*	Inhibition of metformin uptake			
			IC_50_ (μM) = 1.5 (OCT1), 5.8 (OCT2), 4.4 (OCT3)	-	OCT1-3	
			IC_50_ (μM) = 0.05 (MATE1), 0.5 (MATE2)	-	MATE1-2	[112]
	Ketoconazole	*in vitro*	Inhibition of metformin uptake	-	OCT2, MATE1	[105]
	KO143	*in vitro*	Decreased metformin uptake in IOVs	-	BCRP	[72]
	Metoprolol	*in vitro*	Inhibition of metformin uptake (IC_50_ = 50.2 μM)	-	OCT2	[106]
	Mexiletine	*in vitro*	Inhibition of metformin uptake	-	OCT2	[113]
	MPP^+^	*in vitro*	Inhibition of MPP^+^ uptake (IC_50_ = 3.4 mM)	-	OCT1	[15]
	MPP^+^	*in vitro*	Inhibiton of MPP+ uptake (IC_50_ = 397 μM)	-	OCT2	[110]
	MPP^+^	*in vitro*	Inhibition of MPP^+^ uptake (IC_50_ = 2.9 mM)	-	OCT3	[15]
	Nilotinib	*in vitro*	Inhibition of metformin uptake			
			IC_50_ (μM) = 2.9 (OCT1), >30 (OCT2), 0.3 (OCT3)	-	OCT1-3	
			IC_50_ (μM) = 3.4 (MATE1), 1.8 (MATE2)	-	MATE1-2	[112]
	Probenicide	*in vitro*	Inhibition of metformin uptake	-	OCT2, MATE1	[105]
	Propranolol	*in vitro*	Inhibition of metformin uptake (IC_50_ = 8.3 μM)	-	OCT2	[106]
	Pyrimethamine	*in vitro*	Inhibition of metformin uptake	-	OCT2, MATE1	[105]
	Quinidine	*in vitro*	Inhibition of metformin uptake (IC_50_ = 55 μM)	-	OCT1	[114]
	Ranitidine	*in vitro*	Inhibition of metformin uptake	-	OCT2, MATE1	[105]
	Rapamycin	*in vitro*	Inhibition of metformin uptake	-	MATE1	[105]
	Sitagliptin	*in vitro*	Inhibition of metformin uptake (*K*_i_ = 34.9 μM, 40.8 μM)	-	OCT1, OCT2	[87]
	Trimethoprim	*in vitro*	Inhibition of metformin uptake	-	OCT2, MATE1	[105]
	Verapamil	*in vitro*	Decreased metformin uptake in IOVs	-	P-gp	[72]
Pioglitazone	Estrone-3-sulfate	*in vitro*	Inhibition of E3S uptake	-	OATP1B1, OATP1B3	[74]
Repaglinide	Atorvastatin	*in vivo*	AUC of Repaglinide ↑ 1.2-fold	-	OATP1B1	[94]
	Clarithromycin	*in vivo*	AUC of Repaglinide ↑ 40%	CYP3A4	OATP1B1	[49]
	Cyclosporin A	*in vivo*	AUC of Repaglinide ↑ 2.4-fold	CYP3A4	OATP1B1	[52]
	FMTX	*In vitro*	Inhibition of FMTX uptake (IC_50_ = 1.1 μM, 4.8 μM)	-	OATP1B1, OATP1B3	[115]
	Gemfibrozil	*in vivo*	AUC of Repaglinide ↑ 8.1-fold	CYP2C8	OATP1B1	[50,93]
	Pravastatin	*in vitro*	Inhibition of pravastatin uptake	-	OATP1B1, OATP1B3	[64]
	Rifampin	*in vivo*	AUC of Repaglinide ↓60%	CYP3A4	-	[116]
	Telithromycin	*in vivo*	AUC of Repaglinide ↑ 77%	CYP3A4	OATP1B1	[51]
Rosiglitazone	Calcein	*in vitro*	Inhibition of calcein efflux	-	P-gp	[76]
	Estrone-3-sulfate	*in vitro*	Inhibition of E3S uptake	-	OATP1B1	[74]
	Metformin	*in vitro*	Inhibition of rosiglitazone uptake in IOVs (IC_50_ = 0.6 μM)	-	P-gp	[72]
	PhA	*in vitro*	Inhibition of PhA efflux	-	BCRP	[76]
	PhA	*in vitro*	Inhibition of PhA efflux (IC_50_ = 25 μM)	-	BCRP	[71]
	Pravastatin	*in vitro*	Stimulation^b^ and inhibition of pravastatin uptake	-	OATP1B1, OATP1B3	[64]
	Verapamil	*in vitro*	Decreased rosiglitazone uptake in IOVs	-	P-gp	[72]
Sitagliptin	Cimetidine	*in vitro*	Inhibition of cimetidine uptake (IC_50_ = 160 μM)	-	OAT3	[88]
	Cimetidine	*in vitro*	Inhibition of sitagliptin uptake (IC_50_ = 79 μM)	-	OAT3	[88]
	Cyclosporin A	*in vitro*	Inhibition of sitagliptin uptake (IC_50_ = 1 μM)	-	P-gp	[88]
	Fenofibric acid	*in vitro*	Inhibition of sitagliptin uptake (IC_50_ = 2.2 μM)	-	OAT3	[88]
	Furosemide	*in vitro*	Inhibition of sitagliptin uptake (IC_50_ = 1.7 μM)	-	OAT3	[88]
	Ibuprofen	*in vitro*	Inhibition of sitagliptin uptake (IC_50_ = 3.7 μM)	-	OAT3	[88]
	Indapamide	*in vitro*	Inhibition of sitagliptin uptake (IC_50_ = 11.2 μM)	-	OAT3	[88]
	MPP^+^	*in vitro*	Inhibition of MPP+ uptake (*K*_i_ = 34.4 μM, 44.7 μM)	-	OCT1, OCT2	[87]
	Probenicid	*in vitro*	Inhibition of sitagliptin uptake (IC_50_ = 5.6 μM)	-	OAT3	[88]
	Quinapril	*in vitro*	Inhibition of sitagliptin uptake (IC_50_ = 6.2 μM)	-	OAT3	[88]
	Salycylate	*in vitro*	Inhibition of salycylate uptake	-	OAT3	[87]
Troglitazone	Calcein	*in vitro*	Inhibition of calcein efflux	-	P-gp	[76]
	Estradiol-17ß-glucoronide	*in vitro*	Inhibition of E-17ß-G uptake (*K*_i_ = 1 μM)	-	OATP1B1	[117]
	Estradiol-17ß-glucoronide	*in vitro*	Inhibition of E-17ß-G uptake (IC_50_= 1.2 μM, 15.7 μM)	-	OATP1B1, OATP1B3	[75]
	Estrone-3-sulfate	*in vitro*	Inhibition of E3S uptake	-	OATP1B1	[74]
	GCDC	*in vitro*	Inhibition of GCDC transport (IC_50_ = 24.4 μM)	-	BSEP	[77]
	Glycocholate	*in vitro*	Inhibition of glycocholate transport (IC_50_ = 16 μM)	-	BSEP	[77]
	PhA	*in vitro*	Inhibition of PhA efflux	-	BCRP	[76]
	Taurocholate	*in vitro*	Inhibition of taurocholate transport (IC_50_ = 9.5 μM)	-	BSEP	[77]
	Taurocholate	*in vitro*	Inhibition of taurocholate uptake	-	NTCP	[118]
	Taurocholate	*in vitro*	Decreased biliary excretion of taurocholate	-	BSEP	[118]
	TCDC	*in vitro*	Inhibition of TCDC transport (IC_50_ = 27.6 μM)	-	BSEP	[77]
Troglitazone Sulfate	Estrone-3-sulfate	*in vitro*	Inhibition of E3S uptake	-	OATP1B1, OATP1B3	[74]

a Dual perfusion system of isolated human placental lobules;

b Stimulation at low rosiglitazone concentration (10 μM); Abbreviations: 5-CFDA, 5-carboxy fluorescein diacetate; 8-FcA, 8-fluorescein-cAMP; AUC, area under the concentration time curve; BCRP, breast cancer resistance protein; BSEP, bile salt export pump; c_max_, maximum peak concentration in plasma; E3S, estrone-3-sulfate; E17βG, estradiol-17β-glucoronide; FMTX, fluorescein-methotrexate; GC, glycocholate; GCDC, glycochenodeoxycholate; IOV, inside-out placental brush border membrane vesicles; KO143, BCRP-selective inhibitor (Pyrazino[1′,2′:1,6]pyrido[3,4-b]indole-3-propanoicacid, 1,2,3,4,6,7,12,12a-octahydro-9-methoxy-6-(2-methylpropyl)-1,4-dioxo-,1,1-dimethylethyl ester, (3S,6S,12aS)-); MATE, multidrug and toxin extrusion protein; MPP^+^, 1-methyl-4-phenylpyridinium; MRP, multidrug resistance protein; NTCP, sodium-taurocholate cotransporting polypeptide; OATP, organic anion transporting polypeptide; OCT, organic cation transporter; PhA, pheophorbide A; P-gp, P-glycoprotein; TCDC, taurochenodeoxycholate.
